# Evaluating the clinical effectiveness of repeat screening for gestational diabetes mellitus following a diagnosis of large for gestational age and/or polyhydramnios

**DOI:** 10.1002/pmf2.70209

**Published:** 2026-01-07

**Authors:** H. Tal Lesser, A. D. Mackeen, David Chromey, Amanda J. Young, Celia Gray, Michael J. Paglia

**Affiliations:** ^1^ Department of Obstetrics and Gynecology Geisinger Danville Pennsylvania USA; ^2^ Biostatistics Core Department of Population Health Sciences Geisinger Danville Pennsylvania USA; ^3^ Department of Population Health Sciences Phenomic Analytics & Clinical Data Core Geisinger Danville Pennsylvania USA

**Keywords:** gestational diabetes screening, large for gestational age, polyhydramnios

## Abstract

**Objective:**

Gestational diabetes mellitus (GDM) screening is often repeated in the third trimester when large for gestational age (LGA) and/or polyhydramnios are diagnosed. We evaluated outcomes for individuals who did versus did not rescreen, and also for individuals who passed or did not pass rescreening.

**Methods:**

A retrospective cohort study from January 2011 to March 2024 of term, singleton pregnancies diagnosed with LGA and/or polyhydramnios after a normal GDM screen at 24–30 weeks was performed. Non‐inferiority analyses were performed for the comparison between those who did not rescreen and those who did rescreen (passed or did not pass) using a mixed model. In non‐inferiority analyses, if the lower bound of a one‐sided 95% confidence interval (CI) is within the non‐inferiority limit (margin of error), the test is significant, allowing non‐inferiority to be declared. The lower bound of the 95% CI and corresponding *p* values were reported. Outcomes in individuals who passed and who did not pass rescreening were compared using a logistic regression model. Odds ratios with respective 95% CI and *p* values are reported.

**Results:**

Of the 1665 pregnant individuals who met the inclusion criteria, 1317 did not rescreen and 348 rescreened. In individuals who were rescreened, 13.8% were diagnosed with GDM, of which 20.8% required medication. Outcomes were not worse for those who did not rescreen than for those who did rescreen, including birthweight (BW) (3758 vs. 3813 g, −123.65, *p* < 0.01), BW ≥ 4000 (31.9% vs. 34.1%, −0.32, *p* < 0.01), cesarean delivery (21.0% vs. 28.0%, 3.15, *p* < 0.01), or shoulder dystocia (5.2% vs. 6.6%, −1.47, *p* < 0.01). Outcomes for individuals who did rescreen did not differ whether they passed or failed, aside from an increase in neonatal hypoglycemia (7.0% vs. 22.9%, 3.95 [95% CI, 1.76–8.84], *p* < 0.01).

**Conclusion:**

Pregnant individuals who did not rescreen for GDM following the diagnosis of LGA and/or polyhydramnios in the third trimester do not appear to have worse birth outcomes than those who did rescreen. Additionally, individuals diagnosed with GDM after rescreening were not at an increased risk for adverse perinatal outcomes. This study demonstrates that rescreening all individuals with LGA and/or polyhydramnios may not be warranted.

## 1 INTRODUCTION

Despite previous normal results in the second trimester, some providers recommend repeating gestational diabetes mellitus (GDM) screening in the third trimester when large for gestational age (LGA) and/or polyhydramnios are diagnosed due to concern for late onset GDM [1, 2, 3]. Rescreening is likely performed in an attempt to intervene and obviate the increased risks associated with untreated GDM, including hypertension in pregnancy, increased birthweight (BW), birth trauma, and cesarean delivery (CD) [3]. Rescreening is costly, and it is unclear whether intervention is advantageous when GDM is diagnosed in the third trimester [1, 2, 4, 5]. Therefore, rescreening is of uncertain clinical benefit.

Current studies suggest a 10%–20% detection rate for GDM in individuals with abnormal ultrasound findings of LGA and/or polyhydramnios, and of these individuals, approximately 10%–20% require pharmacologic treatment [3]. However, these studies were heterogeneous regarding the two‐step versus one‐step GDM screen and most studies were non‐US‐based, where GDM and obesity are more common [6]. Additionally, these studies focused on outcomes in individuals who did rescreen (passed or did not pass) and not on whether rescreening was done. According to these studies, subjects with GDM had higher rates of shoulder dystocia and CD but no differences in BW. The study aimed to determine whether foregoing repeat testing is associated with worse perinatal outcomes.

## 2 METHODS

We performed an institutional review board–approved, retrospective cohort study from January 1, 2011 to March 1, 2024. This study examined term, singleton pregnancies with a normal GDM screen between 24 and 30 weeks' gestation that were later diagnosed with LGA (estimated fetal weight [EFW] on ultrasound >90% using Hadlock) and/or polyhydramnios (either amniotic fluid index >24 cm or maximum vertical pocket ≥8 cm), and that delivered within Geisinger, a large healthcare system in Central Pennsylvania that supports both rural and urban regions. A diagnosis of LGA and/or polyhydramnios came at the time of an otherwise medically indicated ultrasound. In 2011, the Department of Maternal‐Fetal Medicine (MFM) at Geisinger adopted a policy to recommend rescreening for GDM in pregnant individuals with LGA and/or polyhydramnios if ≥4 weeks had passed since the initial screen. A normal GDM screen was defined using the two‐step method with either a 1‐h 50‐g glucose challenge test (GCT) result <130 mg/dL or abnormal GCT with subsequent normal 3‐h 100‐g oral glucose tolerance test (OGTT) using Carpenter and Coustan cutoff values [6].

Subjects were considered to have rescreened if they completed either the repeat 1‐h GCT and repeat 3‐h OGTT or only the repeat 3‐h OGTT. Individuals who opted for at‐home blood glucose monitoring with fasting and postprandial finger capillary evaluation were not included. Rescreening was not performed if the individual had a normal initial screen within 4 weeks of the abnormal ultrasound. Individuals with pregestational diabetes mellitus, fetal growth restriction, or fetal anomalies known to contribute to abnormal amniotic fluid indices (i.e., tracheoesophageal fistula, complex congenital heart defect, significant urinary tract abnormality, central nervous system abnormality, cleft lip/palate, confirmed/suspected fetal aneuploidy, etc.) were excluded [7].

Data for study subjects were abstracted using previously validated algorithms designed by study authors (A.D.M. and C.G.) [8]. Manual chart review was performed to capture data unable to be extracted programmatically from the electronic health records, including fetal anomalies and indications for CD. For individuals with multiple pregnancies during the study period, only the first pregnancy was included, and a history of prior outcomes was controlled for in the analyses.

The primary outcome was the difference in BW for neonates born to individuals who did not complete rescreening versus those who did. It was hypothesized that in individuals who were diagnosed with LGA and/or polyhydramnios, there would be ≤5% difference in BW (average of 3400 g at term) between those who did not complete rescreening and those who did, regardless of the outcome of the test [9]. Secondary outcomes included BW ≥ 4000 g, obstetric anal sphincter injuries, shoulder dystocia, uterotonic medication use, maternal blood transfusion, wound complications, hypertensive disorders of pregnancy, induction of labor, CD, stillbirth, neonatal intensive care unit (NICU) admission (after assessment from a neonatologist from Geisinger), and neonatal hypoglycemia (defined as blood glucose <40 mg/dL within first 24 h of life requiring intravenous or oral glucose). The indication for CD was classified as macrosomia‐related and non‐macrosomia‐related.

Individuals were grouped into those who did not rescreen and those who did rescreen. Those who did complete rescreening were then further divided into those who passed versus did not pass. For each of the groups, frequencies and percentages were used to describe categorical variables. Means and standard deviations or medians and interquartile ranges (IQRs) were used for continuous variables. Comparisons of maternal and neonatal characteristics between those who did not rescreen and those who did were performed using the chi‐square tests for categorical variables and the *t*‐test or the Kruskal–Wallis tests for continuous variables, as appropriate. *p* values less than 0.05 were considered significant.

Non‐inferiority analyses were performed for the comparison between those who did not rescreen and those who did rescreen (passed or did not pass) using a mixed model. In non‐inferiority testing, the null hypothesis posits that one strategy, in this case, not rescreening, is worse than the alternative (rescreening) by more than a pre‐specified margin of clinical acceptability. The alternative hypothesis asserts non‐inferiority, meaning that outcomes for the non‐rescreened group are not worse than those for the rescreened group beyond that margin. If the lower bound of a one‐sided 95% confidence interval (CI) for the difference in outcomes lies above the non‐inferiority margin (margin of error), the null hypothesis can be rejected, and non‐inferiority is declared. In other words, a *p*‐value < 0.05 indicates statistical significance in favor of non‐inferiority, supporting the conclusion that the group that did not rescreen did not have worse outcomes than the group that did rescreen. Conversely, a *p* value > 0.05 suggests that the data do not provide sufficient evidence to rule out the possibility that the non‐rescreening strategy is inferior, and non‐inferiority cannot be concluded. BW was adjusted for gestational age (GA), history of LGA, and history of GDM. Obstetric and sphincter injury, induction of labor, and neonatal hypoglycemia were adjusted for the history of LGA. Shoulder dystocia was adjusted for the history of LGA and the history of GDM. The lower bound of the 95% CI and corresponding *p* values were reported.

Comparisons between those who passed and did not pass rescreening were performed using a logistic regression model. BW was adjusted for GA, the history of LGA, and the history of GDM. Obstetric and sphincter injury, induction of labor, and neonatal hypoglycemia were adjusted for the history of LGA. Shoulder dystocia was adjusted for the history of LGA and the history of GDM. Odds ratios and respective 95% CI and *p*‐values are reported. All analyses were performed using SAS v9.4 (SAS Institute, Inc.).

## 3 RESULTS

**FIGURE 1:**
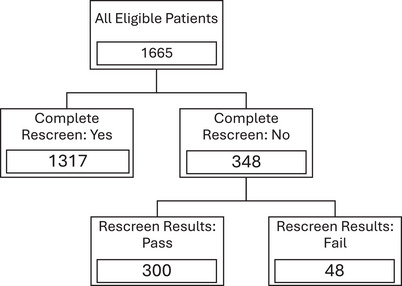
Cohort diagram.

As seen in Figure 1, over the study period, a total of 1665 pregnancies met the inclusion criteria, of which 1317 did not rescreen and 348 did rescreen (300 passed and 48 did not pass). Table 1 depicts baseline characteristics as well as rescreening information with select maternal outcomes for the study population. The median GA of rescreening was 35 weeks (IQRs 33 and 37). In the entire study population, 48.0% had obesity, of which 27.9% had class III obesity, and there were no differences in the class of obesity. A total of 4.7% had a history of an LGA newborn. Though only a total of 3.8% had a history of GDM, this was significantly increased in the group that did rescreen. A total of 24.5% of individuals had an abnormal 1‐h GCT on initial screening and required a 3‐h OGTT. LGA was the more common indication for rescreening as compared to polyhydramnios (44.8% vs. 37.1%). Primary CD for suspected macrosomia occurred infrequently: 5.1% in those who did not rescreen and 3.2% in those who did rescreen.

**TABLE 1:** Patient demographics, clinical characteristics, rescreening information, and maternal outcomes by complete rescreen status and results of completed rescreen.

	Complete rescreen			Results of completed rescreen		
	No (*N* = 1317)	Yes (*N* = 348)	*p* value	Pass (*N* = 300)	Fail (*N* = 48)	*p* value
**Patient demographics and clinical characteristics**						
Age (years), mean (SD)	29.4 (5.32)	29.5 (5.55)	0.81^a^	29.4 (5.49)	29.9 (5.97)	0.39^a^
Race			0.06^b^			0.83^b^
Black or African American	68 (5.2)	11 (3.2)		10 (3.3)	1 (2.1)	
White	1208 (91.7)	3332 (95.5)		286 (95.4)	46 (95.8)	
Other^c^	41 (3.1)	5 (1.3)		4 (1.3)	1 (2.1)	
Pregravid BMI (kg/m^2^), median (IQR)	29.5 (24.8, 35.8)	29.9 (25.1, 36.6)	0.38^a^	29.9 (25.1, 36.2)	31.0 (26.0, 38.5)	0.31^a^
Missing	100	12		9	3	
Pregravid BMI ≥ 30 (kg/m^2^)	583 (47.9)	163 (48.5)	0.84^b^	140 (48.1)	23 (52.3)	0.71^b^
Missing	100	12		9	3	
History of LGA	58 (4.4)	20 (5.7)	0.30^b^	17 (5.7)	3 (6.3)	0.87^b^
History of GDM	43 (3.3)	20 (5.7)	0.03^b^	11 (3.7)	9 (18.8)	<0.01^b^
Chronic hypertension	76 (5.8)	15 (4.3)	0.29^b^	13 (4.3)	2 (4.2)	0.96^b^
**Rescreening information and maternal outcomes**						
Gestational age at rescreening	NA	35 (33, 37)	NA	35 (33, 37)	35 (34, 37)	0.83^b^
Initial screen: Abnormal 1‐h GTT, normal 3‐h GCT	332 (25.2)	77 (22.1)	0.23^b^	53 (17.7)	24 (50.0)	<0.01^b^
**Indication for rescreening**			<0.01^a^			0.99^b^
Large for gestational age and polyhydramnios	112 (8.5)	63 (18.1)		54 (18.0)	9 (18.8)	
Large for gestational age only	782 (59.4)	156 (44.8)		135 (45.0)	21 (43.8)	
Polyhydramnios only	423 (32.1)	129 (37.1)		111 (37.0)	18 (37.5)	
Excessive gestational weight gain	775 (63.7)	232 (69.0)	0.07^b^	200 (68.7)	32 (71.1)	0.75^b^
Missing	100	12		9	3	
**Indication for cesarean**			0.74^b^			0.12^b^
Macrosomia‐related cesarean	136 (25.8)	42 (27.1)		33 (24.8)	9 (40.9)	
Abnormal second stage	99 (72.8)	32 (76.2)		25 (75.8)	6 (66.7)	
Suspected macrosomia	27 (19.9)	5 (11.9)		4 (12.1)	1 (11.1)	
EFW ≥ 5000 g non‐diabetic	2 (1.5)	2 (4.8)		2 (6.1)	0 (0)	
EFW ≥ 4500 g diabetic	0 (0)	2 (4.8)		0 (0)	2 (22.2)	
History of shoulder dystocia	8 (5.9)	2 (4.8)		2 (6.1)	0 (0)	
Non‐macrosomia‐related cesarean	392 (74.2)	113 (72.9)		100 (76.7)	13 (59.1)	
Repeat cesarean	193 (49.2)	48 (42.5)		39 (39.0)	9 (69.2)	
Abnormal first‐stage labor	69 (17.6)	27 (23.9)		27 (27.0)	0 (0)	
Fetal intolerance	64 (16.3)	28 (24.8)		25 (25.0)	3 (23.1)	
Fetal malpresentation	42 (10.7)	6 (5.3)		5 (5.0)	1 (7.7)	
Prime for non‐macrosomia	24 (6.1)	4 (3.5)		4 (4.0)	0 (0)	
Gestational age at delivery, mean (SD)	39.5 (1.00)	39.3 (0.94)	<0.01^a^	39.4 (0.92)	39.0 (0.99)	0.01^a^

*Note*: Data are described using frequencies and percents unless otherwise noted, *n* (%).

Abbreviations: BMI, body mass index; EFW, estimated fetal weight; GCT, glucose challenge test; GDM, gestational diabetes mellitus; GTT, glucose tolerance test; IQR, interquartile range; LGA, large for gestational age; SD, standard deviation.

^a^ Kruskal–Wallis *p*‐value.

^b^ Chi‐square *p*‐value.

^c^ Other races include Asian, Native Hawaiian or other Pacific Islander, and multiple races.

For subjects who rescreened, 13.8% (48/348) were diagnosed with GDM. Characteristics were similar across each group, but individuals who were diagnosed with GDM were more likely to have a history of GDM (18.8% vs. 3.7%, *p* < 0.01), and were also more likely to have had an abnormal 1‐h GCT and require a 3‐h OGTT for their initial screen (50.0% vs. 17.7%, *p* < 0.01).

In Table 2, results for the adjusted non‐inferiority analysis of maternal and neonatal outcomes for individuals who did not complete rescreening (non‐rescreen) versus those who did rescreen are shown. It was significant that compared to those who did rescreen, individuals in the non‐rescreen group were not more likely to have ≥5% difference in mean BW (3758.8 g vs. 3813.6 g, −123.7, *p* < 0.01) or BW ≥4000 g (31.9% vs. 34.1%, −0.32, *p* < 0.01). Individuals in the non‐rescreen group had comparable rates to those who did rescreen for preeclampsia (3.6% vs. 5.3%, −0.21, *p* < 0.01), induction of labor (52.4% vs. 56.1%, −1.00, *p* < 0.01), CD (21.0% vs. 28.0%, 3.15, *p* < 0.01), or shoulder dystocia (5.2% vs. 6.6%, −1.47, *p* < 0.01). For neonatal hypoglycemia, there was an absolute difference of nearly 4.6% between the two groups and an adjusted margin of error of 2.15 (5.5% vs. 10.1%, 2.15, *p* < 0.01), but this fell within the established margin of error of 5%, and so for the individuals who did not rescreen their neonates were not at an increased risk. Next, for all individuals who were rescreened, outcomes were stratified by those who passed and those who did not pass as detailed in Table 3. There was an increase in neonatal hypoglycemia for those who did not pass (7.0% vs. 22.9%, 3.95 [1.76–8.84] *p* < 0.01), but no increase in the amount of NICU admissions (7.5% vs. 6.3%, 0.84 [0.24–2.92], *p* = 0.78). For all additional outcomes, there were no significant differences.

**TABLE 2:** Maternal and neonatal outcomes for all patients by no repeat screen versus rescreen.

	Complete rescreen				
	No (event) (*N* = 1317)	Yes (*N* = 348)	Margin of error (%)	Lower bound of one‐sided 95% confidence interval^a^	Non‐inferiority *p* value^a^
Birthweight (g)^b^, mean (SD)	3758.8 (471.8)	3813.6 (476.2)	−170 g	−123.7	<0.01
Missing	33	1			
Birthweight ≥ 4000 g^b^	410 (31.9)	128 (34.1)	−5.0	−0.32	<0.01
Missing	33	1			
OASI tear^c^	27 (3.2)	5 (2.2)	−2.5	−3.16	0.13
Missing	475	125			
Shoulder dystocia^d^	69 (5.2)	23 (6.6)	−2.5	−1.47	<0.01
Uterotonic medication use	154 (11.7)	56 (14.9)	−5.0	0.03	<0.01
Maternal blood transfusion	35 (2.7)	4 (1.1)	−5.0	−0.56	<0.01
Wound complications	4 (0.3)	1 (0.3)	−5.0	−0.56	<0.01
Hypertensive disorders of pregnancy	201 (15.3)	69 (19.8)	−5.0	1.39	<0.01
Preeclampsia	48 (3.6)	20 (5.3)	−5.0	−0.21	<0.01
Gestational hypertension	153 (11.6)	56 (14.9)	−5.0	0.11	<0.01
Induction of labor^c^	690 (52.4)	211 (56.1)	−5.0	−1.00	<0.01
Cesarean delivery^c^	269 (21.0)	97 (28.0)	−5.0	3.15	<0.01
Missing	33	1			
Stillbirth	3 (0.2)	0 (0.0)	−2.5	−0.63	<0.01
NICU admission	98 (7.6)	28 (7.5)	−2.5	−2.71	0.07
Missing	31	1			
Neonatal hypoglycemia^c^	73 (5.5)	38 (10.1)	−5.0	2.15	<0.01

*Note*: Data are described using frequencies and percents unless otherwise noted, *n* (%).

Abbreviations: GDM, gestational diabetes mellitus; LGA, large for gestational age; NICU, neonatal intensive care unit; OASI, obstetric anal sphincter injury; SD, standard deviation.

^a^ Lower bound of the one‐sided 95% confidence interval and respective *p* value are from a least square means estimate from a mixed model using the reported margin of error as the test value.

^b^ Adjusted for gestational age at delivery, history of LGA, and history of GDM.

^c^ Adjusted for history of LGA.

^d^ Adjusted for history of LGA and history of GDM.

**TABLE 3:** Maternal and neonatal outcomes for all patients by results of completed rescreen (pass/fail).

	Results of completed rescreen			
	Pass (*N* = 300)	Fail (event) (*N* = 48)	Odds ratio^a^ (95% confidence interval)	*p* value^a^
Birthweight (g)^c^, mean (SD)	3809.4 (461.9)	3840.9 (562.2)	Not applicable	0.37^b^
Missing	1	0		
Birthweight ≥ 4000 g^c^	100 (33.4)	18 (37.5)	1.21 (0.61–2.38)	0.59
Missing	1	0		
OASI tear^d^	5 (2.6)	0 (0.0)	Not estimable	
Missing	109	16		
Shoulder dystocia^e^	19 (6.4)	4 (8.0)	1.01 (0.31–3.35)	0.98
GDM	0 (0.0)	48 (100)	Not appropriate	
A1GDM		38		
A2GDM		10		
Uterotonic medication use	49 (16.3)	5 (10.4)	0.60 (0.22–1.58)	0.30
Maternal blood transfusion	3 (1.0)	0 (0.0)	Not estimable	
Wound complications	0 (0.0)	0 (0.0)	Not estimable	
Hypertensive disorders of pregnancy	65 (21.7)	4 (8.3)	0.33 (0.11–0.95)	0.04
Preeclampsia	16 (5.3)	1 (2.1)	0.38 (0.05–2.92)	0.35
Gestational hypertension	49 (16.3)	3 (6.3)	0.34 (0.10–1.15)	0.08
Induction of labor^d^	172 (57.3)	25 (52.1)	0.81 (0.44–1.49)	0.50
Cesarean delivery^f^	85 (28.4)	12 (25.0)	1.06 (0.57–1.95)	0.86
Missing	1	0		
NICU admission	22 (7.4)	3 (6.3)	0.84 (0.24–2.92)	0.78
Missing	1	0		
Neonatal hypoglycemia^d^	21 (7.0)	11 (22.9)	3.95 (1.76–8.84)	<0.01

*Note*: Data are described using frequencies and percents unless otherwise noted, *n* (%).

Abbreviations: GDM, gestational diabetes mellitus; LGA, large for gestational age; NICU, neonatal intensive care unit; OASI, obstetric anal sphincter injury; SD, standard deviation.

^a^ Odds Ratio with respective 95% confidence Intervals and *p* value from a logistic regression model.

^b^ Mixed regression model adjusted for gestational age, *p* value reported.

^c^ Adjusted for gestational age at delivery, history of LGA, and history of GDM.

^d^ Adjusted for history of LGA.

^e^ Adjusted for history of LGA and history of GDM.

^f^ Cesarean delivery as an outcome variable excludes patients who had the following indications: repeat cesarean, fetal malpresentation, and prime for non‐macrosomia.

## 4 DISCUSSION

Similar to existing literature, this study population had a 13.8% rate of GDM in the third trimester, of which 21% required pharmacologic intervention [3]. Individuals in the non‐rescreen group were not at a greater risk for increased BW, BW ≥4000 g, preeclampsia, induction of labor, shoulder dystocia, or CD. There was a higher incidence of neonatal hypoglycemia in the group that was rescreened, but the difference was not statistically significant, likely due to increased screening for neonatal hypoglycemia in the group that was rescreened for GDM. NICU admissions were clinically similar between those who did not and did rescreen. These findings suggest that rescreening for GDM may not be beneficial since both groups are still at an increased risk for adverse outcomes and that LGA and polyhydramnios are associated with adverse pregnancy outcomes independent of GDM [6, 10, 11].

To date, there is conflicting data on the utility of rescreening for GDM in the third trimester and, therefore, clinical guidance is limited [3]. Prior studies used a wide variety of screening and diagnostic testing methodology as well as different indications for rescreening [2, 10, 12, 13, 14]. Such studies also focused on outcomes in pregnant individuals who were rescreened and aimed at comparing outcomes between the mode of delivery and shoulder dystocia. These studies did not compare outcomes between groups that rescreened and those that did not [3, 15]. Our study, however, focused on differences in outcomes between groups that were and were not rescreened.

A systematic review and meta‐analysis evaluating results of individuals who did rescreen was performed and found that subjects with GDM had higher rates of shoulder dystocia and CD but no differences in BW, hypertensive disorders of pregnancy, or neonatal hypoglycemia [3]. Several confounding factors exist when considering results from across the current available literature. Each study evaluates a different methodology for rescreening, GA at rescreening, and indication for rescreening. Due to these differences, it is challenging for clinicians to draw absolute conclusions from these results and derive clinical guidance.

This study had several strengths, including that validated algorithms in an obstetric‐specific internal database were used for data abstraction. The sample size is larger than any other single study and spans a period exceeding 10 years. The exclusion criteria removed a number of potential confounding factors. Although data are limited to a single health system, they include five hospitals that serve both a rural‐ and an urban‐centered patient population across Central and Northeastern Pennsylvania. The health system has uniform practice guidelines from a single MFM group, which provides consistency but may limit generalizability.

Despite the strengths of this study, a few limitations are recognized, most notably the retrospective design. There was a surprisingly low rescreen rate despite this practice being recommended to individuals at Geisinger. This may be attributed to provider bias and a lack of discrete clinical evidence in support of this practice. At this institution, patients with a normal GDM screen within 4 weeks of the diagnosis of LGA and/or polyhydramnios are not instructed to rescreen. This subset of patients was included in the analysis with other subjects who did not rescreen, which may confound results. Individuals who monitored blood glucose via capillary checks in place of the 3‐h OGTT were also excluded, which further limited the study population. It is also possible that individuals with LGA and/or polyhydramnios received some degree of counseling regarding diet, exercise, or weight gain, and this cannot be accounted for in this study design. Data for the degree of LGA (and absolute EFW) as well as the severity of polyhydramnios were unavailable. It is acknowledged that this information contributes significantly to the relative risks to pregnancy. Additionally, it is recognized that suspected LGA and/or polyhydramnios may resolve over serial imaging, and these individuals were still included in the study population and analysis.

Until now, prior research indirectly evaluates this clinical dilemma but only focuses on outcomes for those who passed and did not pass rescreening. These findings demonstrate that not rescreening is not associated with an increase in BW or other pregnancy complications and build on evidence that individuals who are diagnosed with GDM in the third trimester after normal initial screening are not at a significantly increased risk. Prospective, larger studies with adequate power comparing outcomes in individuals who rescreen and do not rescreen should be performed and attention should be given to determine the ideal patient, timing, methodology, and indication for rescreening. Additionally, individuals with LGA who are anticipated to be over 4500 g at delivery may benefit the most from rescreening, as studied and proven recommendations for mode of delivery exist at this cutoff. Similarly, patients with a history of GDM or who required a 3‐h OGTT on the initial screen may also represent a higher risk group that would benefit most from rescreening. Based on the study findings, rescreening for GDM only in the setting of LGA and/or polyhydramnios has no significant association with a difference in maternal or neonatal outcomes.

## CONFLICT OF INTEREST STATEMENT

The authors declare no conflicts of interest.

## FUNDING INFORMATION

The authors received no specific funding for this work.
